# Impaired night-time mobility in patients with Parkinson’s disease: a systematic review

**DOI:** 10.3389/fnagi.2023.1264143

**Published:** 2023-11-22

**Authors:** Shengyiwen Li, Qingyang Lin, Yannan Bao, Yichen Feng, Dianyou Li, Chencheng Zhang

**Affiliations:** ^1^Ruijin Hospital, Shanghai Jiao Tong University School of Medicine, Shanghai, China; ^2^Shanghai Jiao Tong University School of Medicine, Shanghai, China; ^3^Shanghai Jiao Tong University School of Biomedical Engineering, Shanghai, China; ^4^Department of Neurosurgery, Center for Functional Neurosurgery, Ruijin Hospital, Shanghai Jiao Tong University School of Medicine, Shanghai, China; ^5^Department of Neurosurgery, RuiJin-Mihoyo Laboratory, Clinical Neuroscience Center, RuiJin Hospital, Shanghai Jiao Tong University School of Medicine, Shanghai, China; ^6^Shanghai Research Center for Brain Science and Brain-Inspired Technology, Shanghai, China

**Keywords:** impaired bed mobility, nocturnal hypokinesia, Parkinson’s disease (PD), movement disorder, nocturnal akinesia

## Abstract

Impaired bed mobility (IBM) is a symptom characteristic of patients having difficulty intentionally moving their bodies during nighttime sleep. IBM is one of the most common nocturnal symptoms of Parkinson’s disease (PD) and may lead to extreme pain and even death; it also increases the burden on the patients’ caregivers. In this systematic review, we included 19 studies involving a total of 1,407 patients with PD to observe the causes, assessment methods, and treatment options for IBM. We conclude that the extent of IBM is positively correlated with the severity of symptoms such as disease duration, dyskinesia and decreased sleep quality in patients with PD, and the evidence implies that IBM may be able to serve as a prodromal feature in the development of PD. IBM probably results from low nocturnal dopamine concentrations, reduced function of the spinal tract, torque problems in the muscles, and aging. Therefore, treatment is mostly based on continuously increasing the patient’s nocturnal dopamine concentration, while deep brain stimulation (DBS) also has a mitigating effect on IBM. Both scales and sensors are commonly used to measure the severity of IBM, the wearable device monitoring and scales being updated makes measurements easier and more accurate. The future of the advancement in this field lies in the use of more family-oriented devices (such as smart phones or watches and bracelets, etc.) to monitor IBM’s symptoms and select the appropriate therapeutic treatment according to the severity of the symptoms to relieve patients’ suffering.

## Introduction

Parkinson’s disease (PD) is a common neurodegenerative disease that mainly affects middle-aged and older adults, affecting an estimated 8.5 million people in 2019.^1^ The number of people with PD is expected to be more than 12 million by 2040, mainly because of the impact of aging (Dorsey et al., 2018). The primary clinical features of PD include movement disorders, such as progressive bradykinesia and resting tremor, and non-motor symptoms, such as autonomic dysfunction, sensory abnormalities, and sleep disorders (Jankovic, 2008). Sleep duration accounts for about one-third of the day, and sleep disorders have an immense impact on the quality of life of patients with PD. As a type of sleep disorder, impaired bed mobility (IBM) refers to the patient’s inability to move their body freely during night sleep, including difficulty in turning over and getting up (Sringean et al., 2016), and especially difficulty with axial movements (Louter et al., 2013; Bhidayasiri et al., 2017). However, most current research on PD centers on daytime motor and non-motor impairments; the important nighttime symptoms of PD have not attracted public attention.

**Figure 1:**
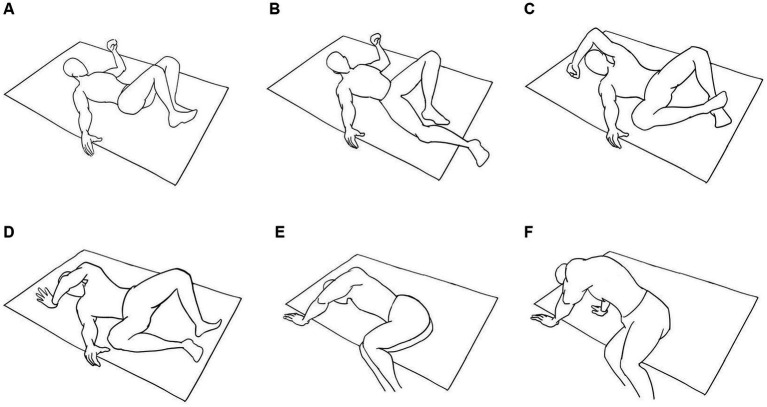
**(A-F)**, Schematic diagrams of wakening difficulties in Parkinson’s disease patients.

Unlike healthy people, patients with impaired bed mobility (IBM) need to get up by grasping the edge of the bed with their hands, and the whole process of getting up takes more steps and time (Figure 1).

The earliest English-language historical reference to similar symptoms of IBM that we have reviewed is Charcot’s lecture on neurological disorders in 1877, stating “patients have the need to change their posture during nighttime sleep, but their limbs are unable to perform the corresponding movements, and prolonged continuation can lead to great pain.” Study in Laihinen et al.’s (1987), generally believed as the earliest systematic study on IBM, showed that patients with PD turned over in bed significantly less frequently than did the control group (Laihinen et al., 1987), a finding supported by studies over the past three decades. IBM was considered as the most troublesome nocturnal symptom in patients with PD (Lees et al., 1988), impacting the quality of life of both patients and caregivers (Viwattanakulvanid et al., 2014), but it is often overlooked by physicians and patients themselves (Shulman et al., 2002; Gallagher et al., 2010). Difficulty in turning and getting up can lead to prolonged supine position in patients with PD (Sommerauer et al., 2015; Sringean et al., 2017; Mirelman et al., 2020). Notably, prolonged fixation in a position during sleep can lead to pressure sores and increased risk of upper airway collapse leading to sleep disordered breathing (SDB), which affects functional outcome and mortality in patients with advanced PD (Matsumoto et al., 2014; Sommerauer et al., 2015).

**Figure 2:**
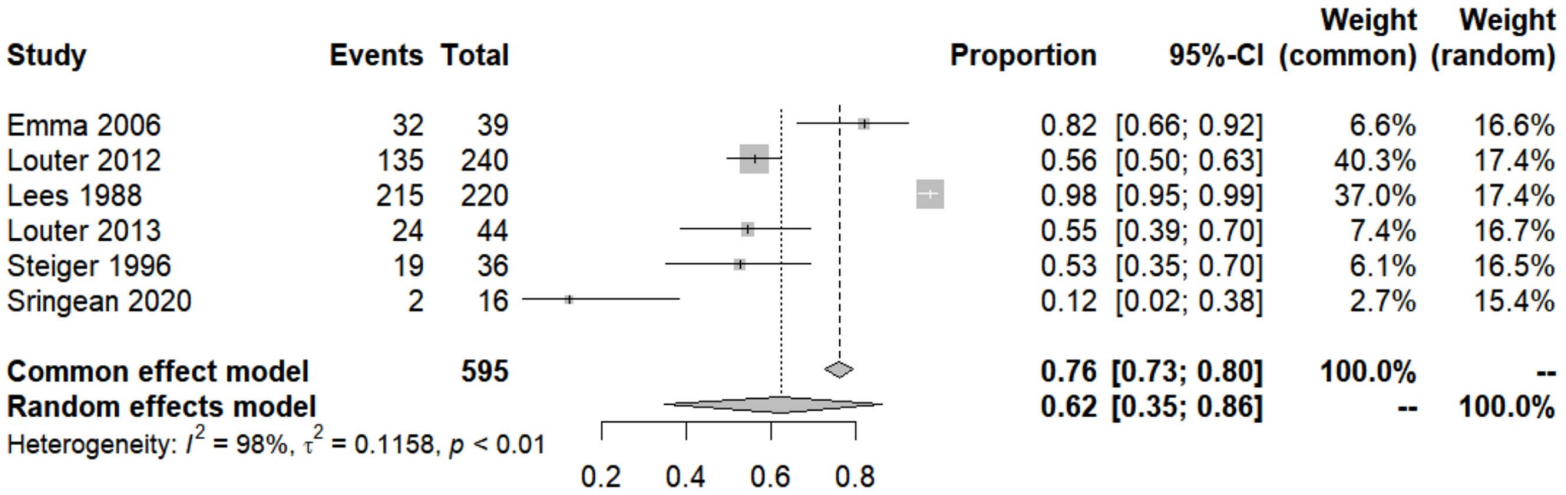
Forest plot of impaired bed mobility (IBM) prevalence among Parkinson’s disease (PD) patients studied by each reference.

In addition to its severity, IBM is also one of the most common symptoms of PD. However, studies are inconclusive regarding the percentage of patients with PD who subjectively experience difficulty turning and getting up, so we summarized the incidence of IBM in PD patients reported in various studies and build a forest plot (Figure 2; Lees et al., 1988; Steiger et al., 1996; Stack and Ashburn, 2006; Louter et al., 2012, 2013; Sringean et al., 2020). We used arcsine transformation to bring the data closer to normal distribution (W = 0.95056, *p* = 0.7448). The test for heterogeneity of the incidence of IBM in PD patients shown in six papers was *Q* = 206.91, *p* < 0.0001, *I*^2^ = 97.6%, which was considered statistically significant. Thus, the random effects model was chosen and the incidence of IBM in PD patients = 0.6241 [0.3486–0.8619]. Possible reasons for the heterogeneity are as follows. Firstly, the publication dates of the six studies span a long period, from 1988 to 2020, and the means of measuring IBM have changed considerably [including oral quizzes, scale assessments (e.g., Question 35 of the Parkinson’s Disease Quality of Life Questionnaire, and the King’s College Hospital (KCH) rating scale), and sensor-based assessments (e.g., NIGHT-Recorder and Actiwatch)]. The development of various unit-dot or multi-dot sensors have improved the accuracy of measuring IBM substantially. While popular commercially available scales for sleep assessment, such as the Pittsburgh Sleep Quality Index (PSQI) and the Medical Outcomes Study Sleep Scale (MOS), have no or only a few questions related to nocturnal bed movement, the emergence of the Nocturnal Hypokinesia Questionnaire (NHQ) in 2018, a scale specifically designed to assess IBM in patients with PD, filled this gap. Secondly, the mean age of the PD patients tested in several articles varied widely, from 55.4 years to over 70 years, and the patients had different Hoehn and Yahr stages. Age and severity of PD may have a huge impact on the prevalence of IBM, so this may also be a source of heterogeneity.

Although the name “impaired bed mobility” or “nocturnal hypokinesia” was widely used to refer to this phenomenon, there is no uniform and authoritative definition of IBM. For example, the definition in Bhidayasiri’s study is “difficulty changing body position in bed (Bhidayasiri and Trenkwalder, 2018),” while the definition in Louter’s study is “PD patients experience issues when turning around in bed and finding a comfortable sleep position (Louter et al., 2013).” Therefore, a systematic definition of IBM symptoms is currently lacking.

A review in 2018 summarized the definition, causes, assessment methods, and treatment options of IBM in patients with PD (Bhidayasiri and Trenkwalder, 2018), and it covers almost all the research related to IBM from 2018 and before. However, as mentioned above, within 5 years after this article was published, there have been very significant developments in both detection and treatment technologies for IBM. In addition, there are some points in this article that have not yet been explained in terms of the reasons for the occurrence of IBM. Therefore, this review aims to add to the previous reviews; address the issues that have not been clearly elucidated by previous studies; and summarize the research on IBM generation mechanisms, detection methods, and treatment options in the last 5 years; the summary will help to further explore the detection and treatment of IBM in patients with PD.

## Methods

### Search method

We searched PubMed, Embase, and Web of Science databases for the period 2015–2022, using “Parkinson,” “PD,” “nocturnal hypokinesia,” “impaired bed mobility,” and “IBM” as keywords in the title and abstract. We retrieved 171 articles in Web of Science, 35 articles in PubMed, and 53 articles in Embase, for a total of 259 articles. After deleting duplicate articles, there were 196 articles.

### Study entry criteria

The retrieved literature was manually browsed to exclude duplicates or literature not related to the study content. Studies were included if the following were indicated in the title and abstract: (1) investigation of clinical and preclinical symptoms of PD-induced nocturnal activity disorder, (2) population consisted of human patients with PD and not animal models of PD, (3) investigation of the mechanism of nocturnal activity disorder initiation in patients with PD, (4) monitoring of nocturnal activity disorder in patients with PD and demonstrated relevance, and (5) description of use of devices to detect nocturnal activity disorders in patients with PD. Non-academic papers were excluded.

In reviewing the selected literature, we found four additional references to the selected literature that were so relevant to the study that they were also included as “from other sources.”

**Figure 3:**
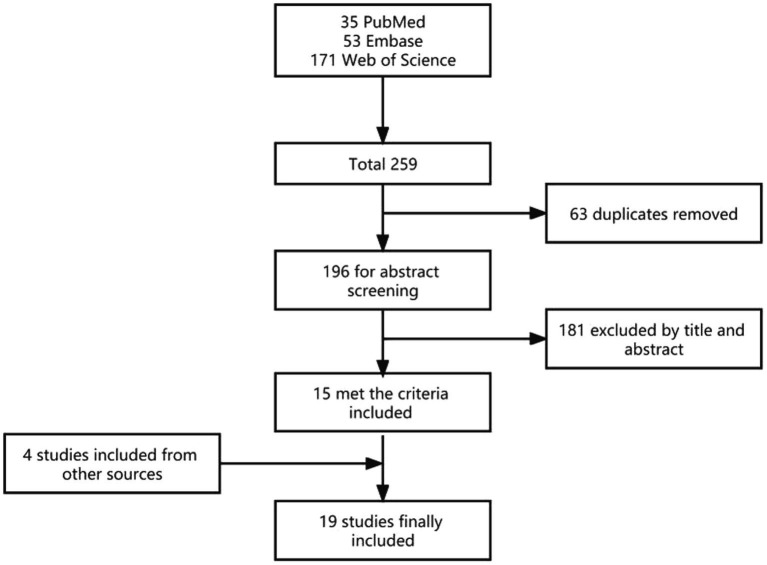
Flow diagram of the literature selection process.

In total, 19 studies involving 1,407 patients with PD, 520 at-risk PD populations, and 478 healthy controls were included in the study in detail (Figure 3).

## Results

### IBM and Parkinson’s disease

#### Correlation between IBM and other symptoms in patients with PD

##### IBM is positively correlated with the duration and severity of PD

Patients with PD with IBM symptoms were older, had longer disease duration, and advanced PD (Sommerauer et al., 2015; Sringean et al., 2020). Patients with Hoehn and Yahr (H&Y) stage 3 PD had increased upright time (time spent sitting, getting up and walking) at night and reduced and slower turning over compared to patients in stage 1 (Mirelman et al., 2020), showing a positive correlation between IBM and the duration of disease and H&Y stage in PD patients (*p* < 0.05, *p* < 0.01).

##### IBM is associated with decreased motor function and sleep status in patients with PD

Patients with mild-to-moderate PD (whose H&Y stage is less than 3) had lower turning efficiency and less frequent and less angular and acceleratory movements compared to their healthy counterparts (Sringean et al., 2016, 2017). The duration of turning and turning ability in patients with PD were significantly correlated with scores on the NHQ and the PD Clinical Scale (PDSS-2) (Sringean et al., 2020). More specifically, IBM and axial dyskinesia were positively correlated in patients with PD (Steiger et al., 1996; Dijkstra et al., 2022). This also supports the idea that turning and getting up are axial movements.

IBM in patients with PD is associated with sleep disturbances (Stack and Ashburn, 2006): in a 2013 study, patients who subjectively reported symptoms of IBM showed fewer sleep-related postural change and reduced sleep efficiency on PSG assessment than PD patients without IBM (Louter et al., 2013). Additionally, the more severe the IBM in patients with PD, the worse the sleep quality (Tandberg et al., 1998; Louter et al., 2012), suggesting that IBM can be used to evaluate nocturnal motor function and sleep status in patients with PD (Xue et al., 2018).

##### Association of IBM with other symptoms in patients with PD

A long duration of PD is independently associated with long supine sleep duration, which is correlated to excessive daytime sleepiness (Sommerauer et al., 2015; Sringean et al., 2020). Moreover, turning disorders are also associated with autonomic dysfunction and gastrointestinal function disorders (Mirelman et al., 2020; Dijkstra et al., 2022). Additionally, IBM also affects the process of waking up the following morning, often referred to as early-morning off, and 60% of patients with PD experience early-morning off almost every morning, with symptoms such as dystonia and a range of non-motor symptoms, including pain, anxiety, and urinary urgency, which can lead to emotional symptoms such as early morning depression (Rizos et al., 2014).

##### IBM can predict whether phenotype switching will occur in at-risk PD group

In a cross-sectional study, Dijkstra et al. showed that subjective tossing disorder is an early symptom of PD and can be used as a basis for prodromal screening in patients predisposed to PD (Dijkstra et al., 2022). In contrast, in another cross-sectional study, Louter et al. showed that nocturnal hypokinesia is a nocturnal manifestation and not a prodromal feature of PD (Louter et al., 2015). Louter et al. also showed that there was no additional turning disorder in the at-risk group (*n* = 33) [who have substantia nigra hyperechogenicity (SN+) as well as have motor sign of PD as assessed by the motor portion of the Unified Parkinson’s Disease Rating Scale (UPDRS-III), and two of the following antecedent/risk markers: prevalence or history of depression, decreased functional capacity, decreased unilateral arm swing, and a positive family history of PD] compared to healthy controls (*n* = 13), leading to the conclusion that IBM is not a precursor feature of PD, whereas the study by Dijkstra et al. compared patients with (*n* = 440) and without (*n* = 47) IBM in the at-risk group [either have an isolated REM Sleep Behavior Disorder (RBD) diagnosed by polysomnography or hyposmia confirmed by the University of Pennsylvania Smell Identification Test (UPSIT)] and showed that IBM predicted the occurrence of phenotypic transition in the at-risk PD group.

A longitudinal study by Fereshtehnejad et al. recruited and annually followed 154 patients with idiopathic RBD confirmed by polysomnography (i.e., at-risk PD group) during 2004–2016 to determine the evolution of multiple motor and non-motor symptoms over time during the prodromal phase of PD, and the results showed that subjects had difficulty turning over in bed between 7 and 11 years before the onset of phenotypic conversion in PD (Fereshtehnejad et al., 2019).

Given the greater reliability of longitudinal studies, combined with differences in sample sizes, we assume that IBM is a predictor of phenotypic transition in the at-risk PD group, but Louter et al.’s findings suggest that IBM may not serve as a highly sensitive screening tool for PD. Further research is required regarding the time frame for the emergence of the prodromal phase of IBM. Because PD cannot be completely cured and can only be relieved by drugs and deep-brain stimulation (DBS), the finding that IBM may be a prodromal symptom of PD could indicate the feasibility of using IBM to predict the onset of PD. With the warning of IBM, clinicians can intervene early in the progression of PD to reduce the degree of functional decline of patients.

#### Causes of IBM

##### Altered dopamine levels

Dopaminergic neuron firing activity rises during non-sleeping hours, and therefore more dopamine is released (Bhidayasiri et al., 2016c), causing a circadian rhythm of high and low dopamine concentrations in the body during day and night, respectively.

The prominent pathological change in patients with PD is the degenerative death of nigrostriatal dopaminergic neurons in the midbrain, resulting in their inability to secrete sufficient amounts of dopamine, a significant reduction in striatal dopamine content, and a loss of storage capacity for dopamine.

To maintain normal dopamine levels, patients with Parkinson’s may choose to take oral dopaminergic drugs regularly during the day. However, levels remain low as these drugs can only be taken once at night before bedtime, resulting in prolonged night periods with no pharmacological intervention, and the dopamine in the body is slowly metabolized.

Additionally, higher doses of dopamine at bedtime have been found to exacerbate insomnia, hallucinations, and dysuria (Sringean et al., 2020); hence, many physicians prefer to avoid nocturnal dopamine therapy. Patients also prefer not to take dopamine at bedtime if their physician does not prescribe it specifically for nighttime symptoms (Bhidayasiri and Trenkwalder, 2018).

Thus, the combination of insufficient dopamine secretion at night, loss of storage capacity, and absence of dopamine intake in patients with PD results in low nighttime dopamine concentrations, which affects muscle activity and impairs nocturnal motor function.

Maintaining a certain level of dopamine throughout the night may improve IBM symptoms in patients with PD (Bhidayasiri et al., 2016c). A number of studies have shown that continuous nighttime dopamine input can improve a range of nocturnal symptoms in patients with PD, as described in later sections.

##### Nervous system dysregulation

PD is a neurodegenerative disease of nerves involved in the motor pathways that control axial movement, including the reticulospinal tract and the vestibulospinal tract (Braak et al., 2003). The reticulospinal tract is involved in the control of muscle movements in the trunk and proximal extremities of the limbs, and the vestibulospinal tract regulates body balance by innervating motor neurons in the extensor and flexor muscles. Although there is no direct evidence that IBM is caused by neurodegeneration, because the function of the reticular and vestibular spinal tracts is associated with turning and waking movements and because PD causes hypofunction of these two spinal tracts, it is reasonable to speculate that hypofunction of the retromedial and vestibular spinal tracts may be a cause of IBM.

One study showed that although patients with PD had impaired axial motor function, they had arm movements significantly more than did their healthy counterparts, with no significant difference in leg movements (Sringean et al., 2016). This result may be because although the reticulospinal tract and vestibulospinal tract are hypofunctional in patients with PD, the function of the corticospinal tract, which controls limb movements (anterior horn motor neurons innervating the upper and lower limbs receive fibers from the lateral corticospinal tract, whereas anterior horn motor neurons innervating the trunk muscles receive fibers from the bilateral corticospinal tract) is not affected. Thus, intact corticospinal tracts ensure normal movement of the extremities and trunk; thus, there is no hypokinesia in the limbs of patients with PD.

Hypofunction of the reticulospinal tract and the vestibulospinal tract leads to axial motor deficits, while the intact corticospinal tract ensures the completion of limb movements; therefore, the symptoms of IBM in PD specifically manifest as axial motor deficits and normal limb movements.

##### Altered muscle function

Impaired bed mobility at night in patients with PD is also associated with a decrease in the ability to use the muscles required to complete turning and waking movements. Although rolling over and waking up is often seen as a simple primary axial rotation movement, rolling over and waking up actually involves many muscle movements and can be divided into multiple steps.

In an earlier study, patients with PD were categorized as using one of three methods of turning over: support by force, sitting up, and using the hips to move (Stack and Ashburn, 2006). There are also three common methods for patients with PD to get up. In particular, the most common way for PD patients to get up is without rotating the upper body (Taniguchi et al., 2022).

One study found that patients with PD had significantly slower movement in bed; significantly lower torque in the hip adductors, abductors, and flexors; and stiff arm muscles compared to healthy controls (Taniguchi et al., 2022). Moreover, several methods of turning and getting up in patients involved supporting and turning the leg with the hand, and turning the leg required the use of the hip adductors. Therefore, reduced torque of the hip adductors on the affected side and arm stiffness on both sides in patients with PD may have contributed to the occurrence of IBM.

##### Effect of aging

Since PD is more prevalent in middle-aged and older populations, age is also a non-negligible factor in the development of IBM; accordingly, aging can contribute to the development of IBM (Stocchi et al., 1998). This is supported by the study by Levy et al., who found a significant correlation between age and motor impairment, axial impairment, and nocturnal bed activity impairment (Levy et al., 2005).

#### Measurement of IBM

##### IBM assessment scale

In 2018, a team in Thailand developed the Nocturnal Hypokinesia Questionnaire (NHQ), an assessment tool specifically designed for nocturnal bed activity disorders, which can be completed independently by a person with PD or a caregiver living with them based on the past week’s experience. The questionnaire has 10 questions in four areas, and all questions are answered with a yes/no response. The severity of the patient’s bed activity impairment is determined by the score (Bhidayasiri et al., 2019).

The NHQ study is limited as it was developed with a small amount of data, had a high proportion of male subjects, and has had a short period of time since development, resulting in fewer studies (two in our collection) using the scale as a reference (Sringean et al., 2020, 2022).

To date, most of the recognized scales have focused more on the nocturnal non-motor symptoms of PD such as insomnia and REM sleep disorders, with only a portion addressing IBM-related questions.

In the most used comprehensive PD scale Movement Disorder Society–Unified Parkinson’s Disease Rating Scale (MDS-UPDRS), only II-9, “In the past week, have you often had difficulty turning over in bed?” relates to bed mobility. Most of the studies used the sum of scores from questions 18, 22, and 27–30 of the MDS-UPDRS-III (called axial scores) to determine patients’ bed mobility (Bhidayasiri et al., 2016a; Xue et al., 2018). Some studies have also used item 9 of the PDSS-2, “Do you feel uncomfortable because you cannot move around in bed?” to determine the presence of IBM (Bhidayasiri et al., 2016a). The quality of life of patients with PD is determined by the PDSS-2. The PD Quality of Life Questionnaire also includes a question related to difficulty turning around in bed (the 35th question: difficulties turning around in bed [Parkinson symptoms]) (de Boer et al., 1996); however, these questions comprise only a small part of the scale, and it is difficult for clinicians to determine whether a PD patient has nocturnal activity disorder or if the disorder is severe based on one or two simple questions.

Compared to the PDSS-2 and UPDRS-III, which ask in general terms whether the patient has difficulty turning at night, the NHQ assesses the IBM in a more detailed and comprehensive way.

##### IBM measurement equipment

###### Polysomnography

Polysomnography (PSG) is the gold standard for the diagnosis of sleep disorders and is defined as the polysomnographic recording of multiple physiological variables during sleep, including those directly and indirectly related to sleep states and stages; PSG is used to assess possible biological causes of sleep disorders (Newman Dorland, 2011).

PSG collects data, such as brain waves, oxygen saturation, heart rate, eye movements, and body position, at night by placing electrodes on the appropriate areas and uploading results to the computer. The PSG system is complete and accurate and can collect specific data according to the needs of the test subject.

However, the disadvantages of PSG are obvious, as detection of data requires a complete set of complex equipment that is expensive to assemble and difficult to complete in the home environment. Additionally, PSG can only be tested in the hospital and can usually only detect night data, which can cause a certain degree of serendipity in the data. Additionally, the PSG electrodes can cause discomfort to patients when worn and fixed, which may indirectly affect the test results.

###### Simple wearable or non-contact monitoring devices

For PD patients, frequent visits to the hospital for PSG monitoring are costly and time-consuming, thus home monitoring devices are better options. In recent years, wearable or non-contact PD monitoring devices for home use have emerged, for which the most obvious advantage is their convenience. A single watch or application can measure multiple values, which not only increases the frequency of measurements and makes it easier for healthcare professionals to observe progress, but also reduces the impact of unfamiliar environments and complex equipment on patients. The following are some of the sensors that have emerged more frequently in current research.

###### Multi-location inertial sensors

The multi-point inertial sensor is used to determine the patient’s movement disorder by measuring the tri-axial acceleration and angular velocity of the patient’s movement, which is generally divided into five sites, two at the wrist, two at the ankle, and one at 10 cm below the fenestra (Xue et al., 2018). The sensor is fixed via nylon straps and is used at a specific frequency (generally 10–20 Hz; Sringean et al., 2016, 2022) for sampling, which is transmitted to electronic devices via Bluetooth or other wireless means.

Multi-point inertial sensors focus on the movement of the patient’s limbs and lumbar rotation to calculate the patient’s turning time, speed, and angle to help the healthcare provider assess the severity of the patient’s difficulty in turning and getting up (Sringean et al., 2022). These sensors are highly specific and can monitor the patient’s movement for a long period of time, especially axial movement, with less impact on the patient’s normal movement and lower cost. Disadvantages include low accuracy and lack of a uniform standard for the devices, making cross-sectional comparison difficult.

As technology continues to advance, more and more home-based, non-contact monitoring devices are becoming available, some of which can monitor how people sleep and turn over at night by placing sensors under the sheets (Takano and Ueno, 2019). If the algorithms of these sensors can be improved to enhance their detection sensitivity, these devices may replace PSG as a convenient and inexpensive new tool for monitoring IBM in the future.

###### Unit point sensors

Other measurement devices used are single-point back acceleration sensors, which are also based on the principle of measuring tri-axial acceleration, with a fixed area in the L4 to 5 region and data capture at 100 Hz (Mirelman et al., 2020). Advantages are lightness, low cost, and also accuracy for gait monitoring (Del Din et al., 2016).

In addition to the one mentioned in the previous paragraph, another type of single-point sensor adds a gyroscope to the accelerometer, obtaining a total of six acceleration and angular velocity signals. The signals include three acceleration axes: (1) vertical acceleration, (2) mid-lateral acceleration, and (3) anterior–posterior acceleration (Herman et al., 2014).

The single-point sensor has less impact on patient activity, but it is questionable whether the accuracy of the measured data is the same as that obtained with the multi-point sensor.

We created a table to compare indicators assessed, accuracy/sensitivity and special feature of scales and wearables used to assess IBM (Table 1).

**Table 1:** Comparison of indicators assessed, accuracy/sensitivity and special feature of scales and wearable devices used to assess IBM.

Types of IBM Assessment Tools	Name	Indicators assessed	Accuracy/sensitivity	Special feature
Scales	MDS-UPDRS (Goetz et al., 2008; Makkos et al., 2018)	The MDS-UPDRS involves participation by patients and caregivers for the assessment of several nonmotor and motor experiences of daily living	There are significant ordinal regression models between the evaluated composite scores and the PGI-I (Nagelkerke pseudo-R-square values: 0.343 for a total score of MDSUPDRS, respectively; *p* < 0.05)	Utilizing composite scores may generally provide a broader assessment of PD
	PDSS-2 (Trenkwalder et al., 2011)	Major problems of sleep in PD consist of nocturnal akinesia like insomnia, sleep fragmentation, rapid eye movement sleep behavior disorder (RBD), restless legs syndrome (RLS), hallucinations and other neuropsychiatric disturbances, sleep apnea syndromes, and nocturia	The total score correlated significantly with the MOS “sleep disturbance” scale (*r* = 0.54, *p* < 0.001) and UPDRS IV.A “dyskinesias” (*r* = 0.36, *p* < 0.001). With a few exceptions, significant correlations were observed between the PDSS-2 total score and all convergent sub-scales of UPDRS and MOS used for the assessment of convergent validity	Reflect a greater spectrum of nocturnal disabilities in PD patients
	NHQ (Bhidayasiri et al., 2019)	PD-specific symptoms associated with nocturnal hypokinesia, including difficulty turning in bed, difficulty getting out of bed, and Parkinson’s disease motor symptoms	The correlation between NHQ and PDSS-2 results was moderate (*r* = 0.32, *p* = 0.004), as was the correlation with objective monitoring (number of turns: *r* = −0.41, *p* = 0.04, degree of turn: *r* = −0.44, *p* = 0.02)	Can be completed by either PD patients or caregivers who sleep in the same environment as them (questions are simpler and not interfered with by patient memory problems)
Multi-location inertial sensors	NIGHT-Recorder (Bhidayasiri et al., 2016b)	Symptoms associated with nocturnal hypokinesia (information on movement, body position relative to gravity, and longitudinal axis rotation while lying in bed)	With the full-scale range set to ±8 g, the smallest change the sensor can detect is 2.44 × 10–4 g, or 2.39 mm/s^2	A easy-to-use, portable, and accurate tool specialized in the assessment of nocturnal hypokinesia
	Multiple Wearable Devices (Bai et al., 2016)	Acceleration, angular velocity, and magnetic flux density, among other information, from both wrists, waist, and ankles of both feet, for a total of five wearable devices that can monitor the movement of a patient’s limbs and body rolls	The resolution of acceleration, gyroscope and magnetometer were 0.48 mg/LSB, 0.06°/s/LSB and 0.63μT/LSB, respectively	(1) Not subject to the subjective influence of patients and clinicians, and has higher reliability and sensitivity; (2) can measure the movement status of the whole body at the same time; (3) can monitor and evaluate the patient’s gait, posture and other movement status at the same time over a long period of time and over a wide range of areas
Unit-point sensors	The Axivity AX3 (AX3 User Manual, n.d.)	Acceleration	Acceleration range:±2 g, ±4 g, ±8 g and ± 16 g	Its lightness, low cost, and also accuracy for gait monitoring
	DynaPort Hybrid (Herman et al., 2014)	Acceleration and angular velocity	A triaxial accelerometer (sensor range and resolution ±2 g and ± 1 mg, respectively) a triaxial gyroscope (sensor range and resolution ±100 and ± 0.0069°/s, respectively)	Responds more accurately to axial motion than a simple accelerometer, more suitable for IBM monitoring
	Commercial bracelets/watches	Accelerometer, temperature, heart rate, oxygen saturation, etc.	No. Currently, the main functions are exercise monitoring, heart rate monitoring and sleep staging, and the results obtained from the accelerometer can be utilized in the measurement and evaluation of IBM through algorithm training in the future	Lightweight, inexpensive, highly accessible, comfortable to wear

Since the main symptom of IBM is difficulty in turning over at night, especially with impaired axial movement, the severity of IBM can be evaluated by axial acceleration and angular velocity. Another characteristic of IBM-difficulty in getting up-can be evaluated by checking videos and questioning the patient. Subjective or objective scores can effectively measure IBM. One study in which PD patients and healthy controls were asked to turn in bed from prone to supine position using the NIGHT-Recorder found that patients with PD had a significantly longer turning duration and slower velocity than healthy controls, and that turning parameters of PD patients correlated strongly with their score on clinical rating scales (Sringean et al., 2020).

A variety of easy-to-use wearable devices will comprise the future of IBM monitoring and even PD monitoring of all symptoms. To improve the accuracy of monitoring results, there are two strategies. The first strategy is to improve the accuracy of the device itself: in the process of device development, developers can adopt an active paradigm (such as requiring patients to roll over and collect patient movement data), which can improve the accuracy of the device more than passively collecting all data of patients’ daily scenes. The second strategy is to combine contact and non-contact devices: for example, the combination of single or multi-site sensors and video or acoustic monitoring data can also improve the accuracy.

Moreover, even if the influence of the unfamiliar environment of the hospital on the patient is eliminated, wearable devices can cause tension in the short term and thus cannot be monitored in completely natural conditions. Longer-term monitoring may erase this disadvantage, but the endurance of the device will become a new technical difficulty.

Most of today’s existing monitoring devices assess IBM by measuring the acceleration or axial rotation angle when a patient rolls over, and some commercially available wearable devices (smart bands, watches etc.) have similar functionality, so if the detection sensitivity and algorithms of commercially available devices are improved, it may not be too long before the cost and complexity of detecting IBM is greatly reduced.

As previously elaborated, IBM may be used in the future as a prodromal symptom of Parkinson’s disease to predict the onset of the disease; accordingly, rapid and accurate measurement of bed turning status is necessary. Further optimization and popularization of wearable monitoring devices for home use will allow individuals at risk of Parkinson’s disease to be identified and treated early to avoid further deterioration of the disease.

##### Treatment of IBM

###### Continuous dopaminergic drug delivery

Treatment of nocturnal hypokinesia should focus on maintaining stable dopamine levels throughout the night (Bhidayasiri et al., 2016c). The use of long-acting drugs or continuous short-acting drugs for symptom control has been shown to be feasible and is known as continuous dopaminergic delivery.

###### Intravenous infusion of dopaminergic drugs

Levodopa is a precursor of dopamine and remains the cornerstone of PD management. However, long-term oral therapy leads to fluctuations in levodopa plasma concentrations and is often associated with the development of motor complications, thus limiting its clinical use. Continuous infusion is considered the optimal route of administration for the treatment of patients with PD and motor fluctuations.

Early studies on levodopa showed that levodopa significantly alleviated nocturnal hypokinesia or early morning motor inability (Leeman et al., 1987; Jansen and Meerwaldt, 1988; Pacchetti et al., 1990; Van den Kerchove et al., 1993; Pahwa et al., 1996; Steiger et al., 1996; Stocchi et al., 1998; Zibetti et al., 2013). However, the addition of a single dose of dopaminergic medication at bedtime alone is unlikely to be sufficient to eliminate nocturnal hypokinesia symptoms. Most of the above studies found that as drug concentrations decreased, the drug effect diminished and the number of tosses in the second half of the night decreased significantly. This suggests that continuous delivery of dopaminergic drugs throughout the night is necessary to obtain a sustained therapeutic effect (Table 2).

**Table 2:** Multiple studies to improve nocturnal concentrations in patients with Parkinson’s disease: a cross-sectional comparison.

Research	Experimental design (double-blind/non-double-blind)	Drug name	Dosage	Drug delivery method	Number of patients (*n*)	Average age	Sex (male%)	Duration of disease (years)	H&Y stage	Basis for judging movement disorders (scale/device)	Remarks	Key findings
Stocchi et al. (1998)	Double-blind	Sinemet CR	–	Oral	40	65.6 ± 19.5	Not mentioned	12.3 ± 7.5	2–4	Self-administered severity scales	Before and after comparison	Sinemet CR significantly improved IBM in patients with increased sleep duration, but it had no effect on sleep fragmentation.
Leeman et al. (1987)	Double-blind	Sinemet-Plus	Two pieces	Oral	11	80 ± 5	Not mentioned	-	3–5	Four resistance strain gauges to monitor motion		Whether based on subjective assessments or based on measurement of the number of times patients moved spontaneously in bed via sensors, nighttime levodopa administration produced clinically significant improvements in sleep.
Jansen and Meerwaldt (1988)	Non-double-blind	Madopar HBS	125–500 mg	Oral	15	60	53.3	12	3–5	Subjective patient evaluation	Before and after comparison	At a mean Madopar HBS dosage of 308 mg, most patients with PD^1^ experienced relief from IBM and a significant reduction in the number of nocturnal wakenings.
Pacchetti et al. (1990)	Non-double-blind	Madopar HBS	978 mg/24 h	Oral	25	63	72.0	6	3	CURS Score		The combination of Madopar HBS, and Madopar standard was effective in producing long-term stable response in patients with fluctuating symptoms.
Pahwa et al. (1996)	Non-double-blind	Sinemet 25/100 Sinemet CR 50/200	-	Oral	15	67. 3 ± 9.6	Not mentioned	7.5 ± 5.2	2–3	Collection of heparinized venous blood samples and clinical exercise scores		The clinical effect of standard carbidopa/levodopa (Std-L) as the first dose appeared significantly earlier than with extended-release carbidopa/levodopa (L-CR). There was no significant difference in dyskinesia between the two treatment groups, and the initial morning dose of Std-L alleviated the problem of delayed onset of clinical response often seen with L-CR.
Steiger et al. (1996)	Non-double-blind	Madopar 250 or Sinemet 25/100	A piece of Madopar 250 or two pieces of Sinemet 25/100	Oral	36	55 ± 10	Not mentioned	10.9 ± 6.7	Off state:1–5	KCH Rating Scale	Before and after comparison	Levodopa played a positive role in the treatment of axial sports injuries.
Bhidayasiri et al. (2017)	Double-blind	Rotigotine	10.46 ± 4.63 mg/24 h	Transdermal patch	34	Experimental group: 60.6(9.5) Placebo group: 63.5 (12.5)	Not mentioned	Experimental group: 9.5 (6.0) Placebo group: 8.3 (5.1)	1–4	NIGHT-Recorder records bed activity data UPDRS PDSS-2 NACDS PDQ-8		Rotigotine increased the number and degree of turning in patients with IBM symptoms, but did not affect the speed and acceleration of turning or the number of nighttime wakenings.
Suzuki et al. (2022)	Non-double-blind	Rotigotine	2–4 mg/24 h, increase to 4 mg/24 h after 1 month of treatment	Transdermal patch	15	71.6 ± 5.7	46.7	4.6 ± 3.9	On state:2.8 ± 0.6 off state:3.3 ± 0.8	MDS-UPDRS Parts 3 and 4 PDSS- 2	Before and after comparison	The use of rotigotine reduced patients’ UPDRS-III and IV scores, improved patients’ nighttime sleep disturbances and daytime sleepiness, and also enhanced cognitive function.
Bhidayasiri et al. (2016a)	Non-double-blind	Apomorphine	34.8 ± 6.5 mg per night	Continuous subcutaneous infusion	10	65.4 ± 12.35	Not mentioned	9.6 ± 3.31	On state: 3.25 ± 0.72	UPDRS Part 3 PDSS-2 NADCS	Before and after comparison	Continuous nocturnal injections of apomorphine were effective in improving patients’ nocturnal hypokinetic symptoms, while demonstrating the feasibility of continuous injections while wearing a wearable device.
Fernández-Pajarín et al. (2021)	Non-double-blind	Apomorphine	Study the effect of different doses of apomorphine	Continuous subcutaneous infusion	22	59.4 ± 6.1	50.0	8.7 ± 3.5	-	UPDRS Parts II, III and IV, PDSS-2	Before and after comparison	APO reduced off-time by 70%, which significantly improved motor symptoms in patients with PD; it also improved frontal lobe dysfunction in patients with PD.
Bergquist et al. (2022)	Non-double-blind	DIZ101	Levodopa (10 mg/mL) and carbidopa (1.25 mg/mL): 600 mg/h	Continuous intravenous infusion	20	68.5 (46–77)	24.0	–	1–3	–	–	The plasma concentration of carbidopa in DIZ101 and DIZ102 was 4-fold higher than LCIG, and subcutaneous administration of levodopa/carbidopa solution at a pH of ~5 rapidly maintained plasma levodopa at a sufficiently high level.
		DIZ102	Levodopa (10 mg/mL) and carbidopa (1.25 mg/mL): 800 mg/h	Continuous subcutaneous infusion								
Freire-Alvarez et al. (2021)	Non-double-blind	Levodopa-carbidopa intestinal gel (LCIG)	–	Continuous enteral infusion	63	Oral drug (control) group: 68.7 ± 7.20 LCIG group: 69.3 ± 6.99	Oral drug (control) group: 51.5 LCIG group: 42.9	Oral drug (control) group: 12.77 ± 6.370 LCIG group: 12.67 ± 4.159	–	UDysRS	–	LCIG was more effective than conventional oral medications in the treatment of movement disorders, and it could also improve motor and non-motor symptoms and quality of life.
Zibetti et al. (2013)	Non-double-blind	Duodopa	Levodopa (20 mg/mL) and carbidopa (5 mg/mL)	Continuous enteral infusion	25	69.9 ± 5.8	64.0	12.1 ± 4.1	–	UPDRS Parts III and IV	Before and after comparison	Continuous duodenal injection of levodopa is significantly effective in the long-term treatment of patients with advanced PD, slowing down nocturnal dyskinesia and improving quality of life.

^1^PD refers to Parkinson’s disease.

Recent studies have shown that it is feasible to rapidly achieve high and stable levodopa plasma concentrations with continuous subcutaneous injections of dopaminergic drugs at lower concentrations, which can significantly improve nocturnal symptoms in response to PDSS-2 (Bhidayasiri et al., 2016a; Bhidayasiri and Trenkwalder, 2018; Bergquist et al., 2022); moreover, when continuous delivery of dopaminergic drugs is not feasible, long-acting dopamine agonists may be an alternative option (Bhidayasiri and Trenkwalder, 2018).

A point of concern is that this alternative drug needs to mimic physiological nocturnal dopamine levels (lower than daytime dopamine levels) to avoid the adverse effects associated with high nocturnal dopaminergic stimulation, including insomnia and psychosis (Zhu et al., 2016). Therefore, nocturnal dopamine levels need to be carefully considered in a clinical setting to balance the benefits of improved motor function with the drawbacks of potential adverse effects.

###### Subcutaneous injection of apomorphine

Apomorphine is a potent, long-acting dopamine receptor agonist. Studies have been able to quantitatively demonstrate that after continuous subcutaneous infusion of apomorphine at night, patients with PD have improved sleep quality (Fernández-Pajarín et al., 2021) and nocturnal hypokinesia, as evidenced by a reduction in dystonia (Reuter et al., 1999) and a significant improvement in the number, speed, and degree of rolling over (Bhidayasiri et al., 2016a).

###### Levodopa/carbidopa enteral infusion and subcutaneous injections

In advanced PD, enteral levodopa/carbidopa gel infusion is superior to oral treatment (Freire-Alvarez et al., 2021).

A recent correlational meta-analysis showed that at least one study observed clinically significant improvement in PDSS-2 scores with levodopa/carbidopa gel infusion at 3, 6, 12, 18, and 24 months and clinically significant improvement in ESS scores at 6 and 12 months (Chaudhuri et al., 2022), suggesting that enteral levodopa/carbidopa gel infusion may significantly improve IBM symptoms in patients with PD.

Regarding the device for continuous subcutaneous injection of levodopa/carbidopa, NeuroDerm, a pharmaceutical company in Israel, has developed a small insulin pump-like pump for 24-h subcutaneous injection of ND0612 (a liquid formulation of the combination drug levodopa and carbidopa) ND0612 is in Phase III clinical trials and has been demonstrated to work for dyskinesia in patients with PD; besides, it is also believed to have a therapeutic effect on IBM (Giladi et al., 2015).

###### Rotigotine patch

The rotigotine patch is a non-ergot dopaminergic receptor agonist with a transdermal delivery system that allows for the continuous and stable release of rotigotine over 24 h for sustained dopaminergic administration. The rotigotine patch significantly improves nocturnal and early morning motor symptoms based on significant improvements in nocturnal activity parameters such as the number and degree of turning in bed, when using the continuous delivery of dopaminergic drugs approach; thus, the efficacy of rotigotine has been objectively demonstrated (Bhidayasiri et al., 2017; Suzuki et al., 2022).

###### DBS

Several randomized clinical trials have shown that DBS may be associated with improved nocturnal symptoms, including a trend toward improved overall UPDRS Part II and Part IV scores in the DBS group. These studies did not specifically assess nocturnal symptoms in PD; however, all studies assessed UPDRS Part II and IV, which included an assessment of the patient’s ability to turn in bed and adjust clothing, as well as any sleep disturbances. Although these specific questions were not assessed individually, there was a trend toward improved overall UPDRS Part II and IV scores in the DBS group in each trial (Weaver et al., 2009; Follett et al., 2010; Schuepbach et al., 2013).

In addition to studies assessed with the UPDRS scale, PSQI scores can also reflect patients’ impaired bed mobility. Two studies found that total PSQI scores were significantly lower after DBS surgery than were total preoperative PSQI scores, with a statistically significant before-and-after difference, suggesting that DBS surgery can alleviate sleep disturbances in patients with PD (Iranzo et al., 2002; Monaca et al., 2004).

Although no specific assessments have been performed for issues such as turning in bed, studies have demonstrated that overall sleep in patients with PD shows an improving trend after DBS (Amara et al., 2012; Bargiotas et al., 2017). Consequently, we hypothesized that DBS could also improve IBM in patients with PD. The development and utilization of an updated sleep assessment scale will help establish the specific improvements in nighttime symptoms that may occur in PD.

### Limitation

This review may have some limitations for the following main reasons: the definition and measurement of IBM cannot be standardized across studies, which makes side-by-side comparisons difficult; secondly, there are fewer studies on IBM in Parkinson’s disease, and it is not possible to do a very detailed and systematic analysis of the various specific aspects of IBM (e.g., prevalence, causes, methods of monitoring, treatment options, etc.).

## Conclusion

Impaired bed mobility is a common, difficult-to-resolve, and often overlooked symptom of PD. This review summarizes the association between IBM and PD symptoms, causes, measurement, and treatment of IBM. We believe that the future research concerning impaired bed mobility for patients with PD must be based on more user-friendly sensors, including wearable and non-contact sensors. Since most patients with PD are primarily at home rather than in the hospital, the development of home monitoring devices for IBM can help to more accurately assess the degree of IBM in patients with PD, thus drawing the necessary attention to IBM from patients, caregivers, and doctors. Along with targeted real-time monitoring devices, levodopa and carbidopa continuous infusion pumps are a promising treatment for IBM. While current carbidopa pump studies have focused on relieving dyskinesia in daytime in patients with PD, this miniature pump may help improve sleep pain in patients with PD.

## Author contributions

SL: Writing – original draft. QL: Writing – original draft. YB: Writing – original draft. YF: Writing – original draft. DL: Writing – review & editing. CZ: Writing – review & editing.

## Footnotes

^1^ https://www.who.int/news-room/fact-sheets/detail/parkinson-disease
